# Clinical Outcomes of Sickle Cell Disease Patients With Myocardial Infarction Undergoing Percutaneous Coronary Intervention: A Nationwide Analysis

**DOI:** 10.7759/cureus.69465

**Published:** 2024-09-15

**Authors:** Oluwasegun M Akinti, Jamal C Perry, Temi Ediale, Muzammil Rehman, Henry O Aiwuyo

**Affiliations:** 1 Internal Medicine, Brookdale University Hospital Medical Center, Brooklyn, USA; 2 Medicine, Brookdale University Hospital Medical Center, Brooklyn, USA

**Keywords:** clinical outcomes, heart failure, myocardial infarction, national inpatient sample, nationwide analysis, percutaneous coronary intervention, sickle cell disease

## Abstract

Sickle cell disease (SCD) patients are predisposed to various cardiovascular complications due to the nature and progression of the disease; the clinical outcomes of SCD patients experiencing myocardial infarction (MI) and undergoing percutaneous coronary intervention (PCI) are not well known.

This study aims to explore a comprehensive nationwide analysis of the clinical outcomes in SCD patients who have suffered an MI and subsequently undergone PCI. It also identifies potential complications and compares their outcomes with non-SCD counterparts with the same interventions.

We conducted a retrospective analysis of SCD patients who have suffered an MI and subsequently undergone PCI using the National Inpatient Sample (NIS) database from 2016 to 2020. The primary outcome was mortality, while the secondary outcomes were the average length of stay, comorbid conditions, and cardiovascular outcomes. Logistic, linear, and Poisson regression model analysis applied for outcomes and adjusting co-founders. P-value <0.05 was considered significant.

A total of 775 patients were analyzed for MI who had PCI with SCD, with a mean age of 58±1.06 years. SCD patients exhibited higher rates of comorbidities, including diabetes mellitus (45.81% vs. 37.84%), obesity (23.87% vs. 20.85%), and chronic kidney disease (CKD) (29.03% vs. 17.36%). Heart failure was more common among SCD patients with 34.19% vs. 26.02% in non-SCD patients (OR 1.5, CI 1.1-2.1, p-value=0.02). Other cardiovascular complications such as stroke, ventricular arrhythmias, atrial fibrillation, pulmonary edema, cardiogenic shock, cardiac arrest, and mortality did not significantly differ between SCD and non-SCD (P-values >0.05).

The study observed that SCD patients experienced a significantly higher incidence of heart failure than non-SCD patients. This implies that SCD patients undergoing PCI for MI exhibit distinct clinical outcomes compared to their non-SCD counterparts.

## Introduction

Sickle cell disease (SCD) is a hereditary hemoglobinopathy characterized by sickle-shaped red blood cells, leading to chronic hemolysis and vaso-occlusive episodes [1]. These pathophysiological mechanisms result in chronic hemolytic anemia and red blood cell changes in morphology to sickle shape because of acute hemoglobin polymerization with deoxygenation; this manifests with repeated painful vaso-occlusive crises, endothelial cell dysfunction, inflammation, and tissue ischemia-reperfusion injuries, which predispose SCD patients to various cardiovascular complications, including an elevated risk of myocardial infarction (MI) [2,3]. The microvascular nature of vascular disease in SCD patients can lead to worse outcomes in patients undergoing percutaneous coronary intervention (PCI) for acute coronary syndrome; despite this increased risk, the clinical outcomes of SCD patients experiencing MI and undergoing PCI still lack comprehensive data, thus, the need for further exploration [2,3]. PCI, a cornerstone in managing acute coronary syndromes, involves mechanically restoring blood flow to the affected myocardium [4]. However, the unique hematologic and vascular challenges in SCD patients may influence the efficacy and safety of PCI, warranting a thorough investigation.

This study aims to bridge the knowledge gap by providing a comprehensive nationwide analysis of the clinical outcomes in SCD patients who have suffered an MI and subsequently undergone PCI. By leveraging a large-scale national database, we seek to elucidate these patients' short-term and long-term prognoses, identify potential complications, and compare their outcomes with non-SCD counterparts undergoing similar interventions. This analysis is crucial for optimizing therapeutic strategies and improving the clinical management of SCD patients presenting with acute coronary syndromes.

## Materials and methods

Study design

We conducted a retrospective analysis of heart failure admissions using the National Inpatient Sample (NIS) database from 2016 to 2020.

We queried the NIS between 2016 to 2020. We searched NIS for adult patients hospitalized using the International Classification of Diseases, 10th edition (ICD-10) codes. The primary outcome was mortality, while the secondary outcomes were the average length of stay, comorbid conditions, and cardiovascular outcomes such as heart failure, stroke, ventricular arrhythmia, atrial fibrillation, pulmonary edema, cardiogenic shock, and cardiac arrest. Logistic, linear, and Poisson regression model analysis applied for outcomes and adjusting co-founders.

Database

The NIS is part of the Healthcare Cost and Utilization Project (HCUP) and is maintained by the Agency for Healthcare Research and Quality (AHRQ) [5]. The NIS contains information on all inpatient stays (not individual patients) in 48 states plus the District of Columbia, representing approximately 98% of the United States population, excluding rehabilitation and long-term acute care hospitals [5]. Unweighted, it contains data from more than seven million hospital stays each year, and weighted, it estimates more than 35 million hospitalizations nationally [6]. Observations in the NIS contain a primary diagnosis, up to 39 secondary diagnoses, and up to 25 procedure codes, depending on the year [6]. All discharge diagnoses and procedures were identified using the ICD-10 codes [5,6]. The AHRQ made these data available to the principal author via the HCUP.

Statistical analysis 

National estimates were obtained using the discharge-level weight variable (DISCWT) provided by the HCUP. Weighted data were used for all statistical analyses. Missing values were excluded from the analysis. Categorical variables were compared using the Chi-square test and are described using frequency with percentages. Continuous variables were compared using the student’s t-test and are reported as mean (±SD) if their distribution was normal or compared using the Mann-Whitney U test and reported as median (interquartile range (IQR)) if their distribution was skewed. Multivariable logistic and linear regression analyses were conducted to assess the association with all previously specified endpoints. Variables used in the regression model building were either selected from the dataset as provided variables or abstracted with the ICD-10 codes. The variables used for the regression model include admission year, age, sex, race/ethnicity, primary payer status, socioeconomic stratum, hospital size, hospital location, hospital teaching status, total hospital charge, length of stay, diabetes type 1 and type 2, primary hypertension, hyperlipidemia, PCI, non-ST and ST-elevation MI, ventricular arrhythmias, atrial fibrillation, cardiac arrest, stroke, pulmonary embolism, mortality, and Charleson comorbidity index.

Variables were selected into the multivariate regression model if they were statistically significant (p<0.05) in the univariable regression screening. In addition, we forced well-established variables to affect outcomes based on prior research. Logistic regression results are represented as adjusted odds ratios (ORs) and their respective 95% CIs. Multivariate Linear regression results are expressed as beta coefficients (Coef.) and their respective 95% CIs. Statistical analysis was performed using STATA/BE 18.0 (StataCorp, College Station, TX). P-values <0.05 were considered statistically significant. The checklist for working with the NIS, as recommended by AHRQ, was used to ensure the appropriateness of data analysis [5].

## Results

Baseline characteristics

The baseline characteristics are represented in Table 1. The study analyzed 15590085 non-SCD and 775 SCD patients who had PCI for MI, and the mean age was 64.4±1.31 years vs. 58.00±1.06 years for non-SCD and SCD, respectively. Significant statistical differences existed between the mean age (P<0.001).

**Table 1:** Patient characteristics ^t^Student t-test ^C^Chi-square ^f^Fisher's exact *P-values <0.05 is significant

Variable	Non-SCD (%)	SCD (%)	Statistics	P-value
Age (mean±SD)	64.4± 1.31 years	58.00±1.06 years	18491.730^t^	<0.001*
Gender			10.810^c^	0.001*
Male	1063608 (68.2)	350 (45.2)		
Female	495477 (31.8)	425 (54.8)		
Median household income by zip code			3.989^f^	0.263
0 to 25th percentile (low income)	455253 (29.2)	313 (40.4)		
26th to 50th percentile (median)	432646 (27.7)	221 (28.5)		
51st to 75th percentile	377299 (24.2)	149 (19.2)		
76th to 100th percentile (high income)	293887 (18.9)	92 (11.9)		
Race			111.017^f^	<0.001*
White	1181631 (75.8)	73 (9.4)		
Black	146866 (9.4)	635 (81.9)		
Hispanic	128625 (8.3)	42 (5.4)		
Asian	41627 (2.7)	10 (1.3)		
Native American	9354 (0.6)	0 (0.0)		
Others	50982 (3.2)	15 (2.0)		
Hospital bed size			1.014^c^	0.602
Small	249456 (16.0)	125 (16.1)		
Medium	467724 (30.0)	185 (23.9)		
Large	841905 (54.0)	465 (60.0)		
Hospital metrics				
Length of Stay (days) (mean±SD)	3.6±1.01	4±1.3	72.230^t^	<0.001*
Total charge (USD)	113656.1	118638.4	1.920^t^	0.166
Insurance type			0.978^f^	0.807
Medicare	797160 (51.1)	408 (52.7)		
Medicaid	157935 (10.1)	103 (13.3)		
Private insurance	511692 (32.8)	233 (30.0)		
Uninsured	92298 (6.0)	31 (4.0)		

The non-SCD cohort has 1063608 (68.2%) males and 495477 (31.8%) females while the SCD group has 350 (45.16%) males and 425 (54.84%) females. There was also a significant difference in gender of the two groups (P=0.001).

The racial composition of the non-SCD was mostly white with 1181631 (75.8%); on the other hand, the racial composition of the SCD group was predominantly Black (635, 81.94%), followed by White (73, 9.42%), Hispanic (42, 5.42%), Asian (10, 1.29%), and Other (15, 1.94%). There was also a significant difference in the racial composition of the two groups (P<0.001).

Most patients were treated in large hospitals in both groups with 841905 (54.0%) vs. 465 (60.00%) in non-SCD and SCD respectively, lower percentages in medium bed size with 467724 (30.0%) vs. 185 (23.87%), and small hospital bed size with 249456 (16.0%) vs. 125 (16.13%). There was no significant difference in the hospital bed size of the two groups (P=0.602).

In the non-SCD group, low-income areas accounted for 455253 (29.2%), median areas with 432646 (27.7%), 51st to 75th percentile with 377299 (24.2%), and high-income areas with 293887 (18.9%). The proportion is similar in the SCD group with 313 (40.40%) patients living in the lowest income areas, 221 (28.52%) in the median income range, 149 (19.23%) in the 51st to 75th percentile, and 92 (11.87%) in the highest income range. There was no significant difference in the hospital bed size of the two groups (P=0.263).

Insurance coverage was similar in both groups with Medicare in 797160 (51.1%) vs. 408 (52.67%), followed by private insurance in 511692 (32.8%) vs. 233 (30.06%), Medicaid in 157935 (10.1%) vs. 103 (13.29%), and uninsured in 92298 (6.0%) vs. 31 (4.00%). There were no significant statistical differences between the two groups (P=0.807)

The average length of stay was 3.6±1.01 vs. 4±1.3 days, with a mean total charge of $113656.1 vs. $118,638.40 in non-SCD and SCD, respectively.

Regarding comorbidities, diabetes mellitus was present in 355 (45.81%) of SCD patients compared to 589958 (37.84%) in non-SCD patients; the difference was statistically significant (P<0.001). Obesity was seen in 185 (23.87%) of SCD patients while it was lower with 325069 (20.85%) in non-SCD patients; the difference was also statistically significant (P=0.038). Dyslipidemia was higher in 1106950 (71.01%) patients without SCD compared to 500 (64.52%) with SCD; the difference was statistically significant (P<0.001). Tobacco use was noted in 390 (50.32%) of SCD patients while about the same proportion was noted in 798096 (51.19%) of non-SCD patients; the difference was not statistically significant (P=0.629). Chronic kidney disease (CKD) was higher in 225 (29.03%) SCD patients than in 270657 (17.36%) non-SCD patients; the difference was statistically significant (P<0.001). Hypertension was observed less in 365 (47.10%) SCD patients than in 765043 (49.07%) non-SCD patients; however, the difference was not statistically significant (P=0.272). SCD patients had a higher in-hospital prevalence ≥3 comorbidities (435, 56.13%) compared to patients without SCD (645462, 41.40%), indicating a greater burden of comorbid conditions in the SCD population (see Table 2).

**Table 2:** Comorbidities of patients ^C^Chi-square test ^f^Fisher's exact test *P-values <0.05 is significant CKD, chronic kidney disease; SCD, sickle cell disease

Variables	Non-SCD (%)	SCD (%)	Statistics	P-value
Dyslipidemia	1106950 (71.00)	500 (64.52)	15.815^c^	<0.001*
Diabetes mellitus	589958 (37.84)	355 (45.81)	20.899^c^	<0.001*
Obesity	325069 (20.85)	185 (23.87)	4.284^c^	0.038*
Tobacco use	798096 (51.19)	390 (50.32)	0.233^c^	0.629
CKD	270657 (17.36)	225 (29.03)	73.543^c^	<0.001*
Hypertension	765043 (49.07)	365 (47.10)	1.207^c^	0.272
Charleson comorbidity index			78.276^f^	<0.001*
0	0 (0.00)	0 (0.00)		
1	475520 (30.50)	145 (18.71)		
2	438103 (28.10)	195 (25.16)		
≥3	645462 (41.40)	435 (56.13)		

Analysis of major cardiovascular outcomes

Among patients with SCD, 34.19% experienced heart failure compared to 26.02% of non-SCD patients (OR: 1.5, 95% CI: 1.1-2.1, p=0.02). Stroke occurred in 3.87% of SCD patients and 2.39% of non-SCD patients (OR: 1.6, 95% CI: 0.73-3.71, p=0.2). Ventricular arrhythmias were present in 9.68% of SCD patients versus 11.35% of non-SCD patients (OR: 0.8, 95% CI: 0.5-1.4, p=0.5). Atrial fibrillation was observed in 10.97% of SCD patients and 13.94% of non-SCD patients (OR: 0.8, 95% CI: 0.46-1.26, p=0.2). Pulmonary edema occurred in 1.94% of SCD patients compared to 1.26% of non-SCD patients (OR: 1.5, 95% CI: 0.49-4.85, p=0.4). Cardiogenic shock was noted in 4.52% of SCD patients versus 7.15% of non-SCD patients (OR: 0.6, 95% CI: 0.29-1.31, p=0.2). Cardiac arrest occurred in 5.81% of SCD patients and 3.80% of non-SCD patients (OR: 1.5, 95% CI: 0.8-3.0, p=0.2). Mortality was observed in 2.58% of SCD patients compared to 3.27% of non-SCD patients (OR: 0.95, 95% CI: 0.35-2.6, p=0.9). Intra-aortic balloon pump (IABP) was reported in 3.23% of SCD patients and 4.14% of non-SCD patients (OR: 0.82, 95% CI: 0.34-2.01, p=0.6) (see Table 3).

**Table 3:** Multivariate logistic regression *Statistically significant (P-value <0.05) IABP, intra-aortic balloon pump

Variables	Odd ratio	P-value	95% CI
Heart failure	1.5	0.020*	1.1-2.1
Stroke	1.6	0.200	0.73-3.71
Ventricular arrhythmia	0.8	0.500	0.5-1.4
Atrial fibrillation	0.8	0.200	0.46-1.26
Pulmonary edema	1.5	0.400	0.49-4.85
Cardiogenic shock	0.6	0.200	0.29-1.31
Cardiac arrest	1.5	0.200	0.8-3.0
Mortality	0.95	0.900	0.35-2.6
IABP	0.824664	0.600	0.34 – 2.01

**Figure 1:**
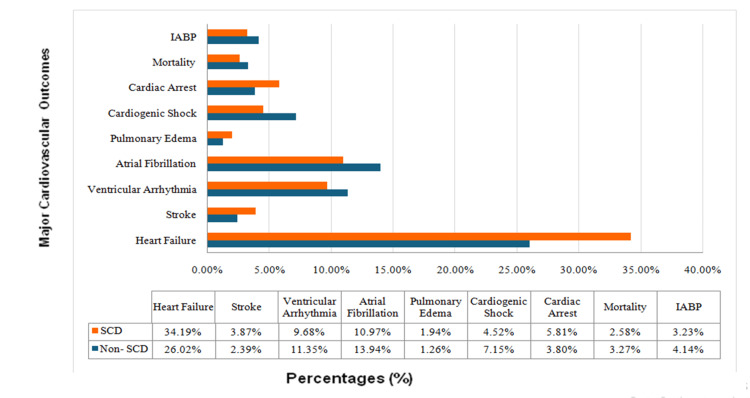
Differences in clinical outcomes between SCD and non-SCD patients IABP, intra-aortic balloon pump; SCD, sickle cell disease

The graph in Figure 1 compares the percentages of various clinical outcomes between patients with SCD and those without (non-SCD), with orange bars representing SCD and blue bars representing non-SCD. It highlights differences in outcomes such as heart failure, stroke, ventricular arrhythmia, atrial fibrillation, pulmonary edema, cardiogenic shock, cardiac arrest, mortality, and the use of IABP support.

## Discussion

The prevalence of MI in patients with SCD is low, ranging from 0% to 3% [2]. This varies with the prevalence in the general population with prevalence as high as 23.3% [7]. Additionally, MI in SCD patients is often due to microvascular alterations rather than atherosclerotic stenosis and can be easily missed due to atypical presentations, diffuse chest pain, and non-specific electrocardiogram changes [2,8]. Most patients with SCD are young and often experience painful crises, so they are often overlooked for MI. MI in other groups also varies from SCD patients with identifiable non-genetic or modifiable factors such as physical inactivity, smoking, alcohol consumption, dyslipidemia, diabetes mellitus, hypertension, and obesity [9,10].

Despite advances in the diagnosis and treatment of coronary artery disease through PCI, there remains evidence of a disparity in the outcomes between patients; the postulated causes for this disparity in PCI outcomes are multifactorial; the factors include but are not limited to atypical presentations and delays in diagnosis [11]. SCD patients are at risk of atypical presentations and delays in diagnosis of MI and as such the index study aimed to determine whether SCD worsens clinical outcomes in patients with MI after PCI. Our finding shows that there was no significant difference in major adverse cardiovascular events (MACE) between SCD and non-SCD patients, except for heart failure. Although SCD patients may exhibit higher rates of comorbidities, including diabetes mellitus, obesity, dyslipidemia, and CKD, indicating a greater burden of underlying health conditions [12,13].

In the index review, heart failure was more common among SCD patients (34.19%) compared to non-SCD (26.02%); this could be attributed to the unique pathophysiology of SCD in addition to the hydration therapy instituted as a mainstay of preventing contrast-induced acute kidney injury (CI-AKI) during PCI [14-16]. Additionally, the incidence of heart failure among SCD patients can also be attributed to chronic anemia from SCD, which may result in cardiac chamber dilation, which progresses to an increase in left ventricular mass and eventual left ventricular diastolic dysfunction [17]. Another notable pathway is through recurrent ischemia, which is complicated [17]. During ischemia, myocardial fibers passive stiffness increases, leading to impaired myocardial relaxation, and then the left ventricular filling pressure increases, further restricting myocardial blood flow, aggravating ischemia, and leading to pulmonary congestion and shortness of breath, which are the hallmarks of heart failure [17]. These factors have been reported to increase the risk of post-PCI acute heart failure (AHF), which is more now prevalent in SCD patients.

The overall incidence of post-PCI AHF reported by Guo et al. was 8.7%; this is very low compared to our index finding both in SCD and non-SCD groups [16]. Similarly, a cohort study by Gu et al. and Cheng et al. reported an incidence of 12.7% and 15.9%, respectively [17,18]. With the increase of the aging population and commensurate increased survival rate after MI with the advent of PCI, the prevalence of HF has reportedly increased, among which HFpEF has become the predominant type. Therefore, newer techniques should be looked into to advert post-PCI AHF.

Recent reports support that post-PCI AHF is a strong predictor of all-cause long-term mortality [19,20]. Additionally, microvascular resistance reserve (MRR), CKD stage, AMI, age, and smoking have been also implicated as independent predictors of all-cause long-term mortality [20-22]. The index review shows no statistical differences in mortality outcomes between SCD patients and non-SCD patients; these ironical outcomes show the importance of timely identification of subjects at risk for HF development and as such early initiation of guideline‐directed HF therapy in these patients; with the institution of these measures, there would be a decrease in the HF burden as well as the reduction in mortality [23].

In SCD patients, cardiac involvement may be initiated from anemia and HbS polymerization, leading to increased cardiac output, peripheral vascular dilation, and volume overload, which result in eccentric hypertrophy and high-output heart failure [3,24]. The higher rate of heart failure among SCD patients is due to a combination of diastolic dysfunction from eccentric remodeling, microvascular obstruction, and repeated vaso-occlusions [2,24]. These vaso-occlusions and hemolysis trigger inflammation and vascular dysfunction, causing chronic organ damage, diffuse myocardial fibrosis, and cardiac remodeling [25]. MI in SCD patients is often caused by microvascular occlusions rather than atherosclerosis, with increased myocardial demand and chronic anemia leading to microvascular ischemia and infarction. Treatment with blood transfusions may exacerbate cardiac dysfunction due to inflammation and iron-mediated injury. While pulmonary hypertension is a concern, it is frequently linked to left heart failure in aging SCD patients [2]. This underscores the importance of close monitoring and appropriate therapeutic interventions for heart failure in SCD patients.

Despite these comorbidities, the rates of complications such as stroke, ventricular arrhythmias, atrial fibrillation, pulmonary edema, cardiogenic shock, cardiac arrest, and mortality did not significantly differ between the two groups. This suggests that while SCD patients have a higher burden of comorbidities, their overall clinical outcomes after PCI for MI are comparable to those without SCD, except for the increased in-hospital prevalence of heart failure.

These findings highlight the importance of tailored treatment approaches and comprehensive care strategies for SCD patients to effectively manage their higher comorbidity burden and improve their overall outcomes. Considering their unique pathophysiological challenges, the findings underscore the need for tailored clinical management strategies for SCD patients with MI; additionally, closer surveillance of these patients cannot be overemphasized. Enhanced understanding and awareness of these risks can guide clinicians in optimizing treatment protocols, ultimately improving prognosis and quality of care for this vulnerable population. Future research should aim to delineate these relationships further and explore targeted interventions to mitigate adverse outcomes in SCD patients undergoing PCI.

Limitations

The limitations of this study include potential coding and documentation errors in administrative databases, which could exclude eligible patients from our analysis. Another limitation is the lack of patient-specific details, such as lab results, imaging findings, medications, and social history. The NIS database does not allow stratification by severity or laboratory values and only includes hospitalized patients, excluding those treated in outpatient settings. The reliance on ICD-10 codes for identifying comorbidities and outcomes may lead to misclassification bias. Additionally, the lack of granular clinical data limits the ability to fully adjust for potential confounders. Despite these limitations, the large sample size and nationwide scope provide valuable insights into the clinical outcomes of SCD patients undergoing PCI for MI. However, our multivariate regression models might have been affected by multicollinearity or other covariates, impacting robustness. Unaccounted variables, potential selection, and unmeasured biases remain a concern, even with multivariable analysis efforts.

## Conclusions

In this nationwide analysis, we observed that SCD patients undergoing PCI for MI exhibit distinct clinical outcomes compared to their non-SCD counterparts. Notably, SCD patients experienced a significantly higher incidence of heart failure, highlighting the considerable cardiovascular burden imposed by SCD. Although the occurrences of stroke, ventricular arrhythmias, atrial fibrillation, pulmonary edema, cardiogenic shock, cardiac arrest, and mortality did not reach statistical significance, the observed trends suggest a complex interplay of factors influencing these outcomes.
